# Prophylaxis after Exposure to *Coxiella burnetii*

**DOI:** 10.3201/eid1410.080576

**Published:** 2008-10

**Authors:** Claire E. Moodie, Herbert A. Thompson, Martin I. Meltzer, David L. Swerdlow

**Affiliations:** Centers for Disease Control and Prevention, Atlanta, Georgia, USA

**Keywords:** Coxiella burnetti, Q fever, prophylaxis, risk-benefit assessment, pregnant women, research

## Abstract

*Coxiella burnetii* is a category B bioterrorism agent. We numerically evaluated the risks and benefits from postexposure prophylaxis (PEP) after an intentional release of *C. burnetii* to the general population, pregnant women, and other high-risk populations. For each group, we constructed a decision tree to estimate illness and deaths averted by use of PEP/100,000 population. We calculated the threshold points at which the number of PEP-related adverse events was equal to the cases averted. PEP was defined as doxycycline (100 mg 2×/day for 5 days), except for pregnant women, where we assumed a PEP of trimethoprim-sulfamethoxazole (160 mg/800 mg 2×/day) for the duration of the pregnancy. PEP would begin 8–12 days postexposure. On the basis of upper-bound probability estimates of PEP-related adverse events for doxycycline, we concluded that the risk for Q fever illness outweighs the risk for antimicrobial drug–related adverse events when the probability of *C. burnetii* exposure is >7% (pregnant women using trimethoprim-sulfamethoxazole = 16%).

## Abstract 2

Postexposure prophylaxis may avert Q fever illness and death when the probability of exposure is above the population-specific threshold point.

Q fever is caused by the intracellular bacterium *Coxiella burnetii* and is endemic in nearly every country in the world. A zoonotic disease, it is usually transmitted to humans through aerosolization of the bacteria from animal products; person-to-person transmission is rare (*1*–*4*).

Roughly 50% of all *C. burnetii* human infections are asymptomatic (*5*–*8*). Acute illness is usually characterized by sudden onset febrile illness; chronic disease occurs in ≈1% of all acute cases with endocarditis being the most common chronic condition (60%–70%) (*1*,*8*–*12*). Persons with preexisting cardiac valve defects are at significantly higher risk for chronic disease; chronic disease develops in 39% of patients treated for acute disease (in 75% without treatment) (*13*–*15*). Immunocompromised patients (e.g., HIV-positive and cancer patients) are also at increased risk for chronic illness.

A Q fever–associated chronic fatigue syndrome may exist as well. Although prevalence is controversial, studies have cited that 10%–30% of all patients with acute disease report persistent symptoms (e.g., fatigue, myalgia, night sweats) more than a year after acute infection occurred (*10*,*16*). Pregnant women are also at increased risk for severe acute *C. burnetii* infection because of the bacterium’s predilection for the placenta. Premature birth (33%) and spontaneous abortion/neonatal deaths (39%) occur frequently in acutely ill pregnant women (*17*).

*C. burnetii* is classified as a category B bioterrorism agent by the Centers for Disease Control and Prevention and the National Center for Allergy and Infectious Diseases (*18*). Regardless of the likelihood that *C. burnetii* may be used as a bioterrorism agent due to its status as a category B agent, public health agencies are obligated to prepare for such a scenario. Current Q fever postexposure prophylaxis (PEP) guidelines for the general population are 100 mg of doxycycline (or 500 mg tetracycline 2×/day for 5 days), started 8–12 days postexposure (*4*). This recommendation is based on limited studies conducted at Fort Detrick, Maryland, USA in the 1950s, which indicated that administering antimicrobial drugs directly after exposure to *C. burnetii* extended the incubation period by 8–10 days but did not prevent infection from occurring (*19*). Waiting 8–12 days after exposure before starting treatment prevented illness (*19*). Unfortunately, these guidelines do not account for the probability of exposure and prophylaxis-related adverse events. Also, the US government has not published any PEP recommendations for pregnant women, although trimethoprim-sulfamethoxazole (TMP-SMX) has been suggested as a possibility (*1*,*4*).

To assist in the development of PEP recommendations, we present a risk-benefit analysis, estimating the number of cases of illness/death that could be averted with PEP after a large release of Q fever versus a treatment-only strategy where antimicrobial drugs are administered only upon symptom onset. We also determine the threshold probability of exposure at which the risk for antimicrobial-related adverse events outweighs the risk for Q fever illness.

## Methods

### Model

In 2006, we conducted a risk-benefit analysis for each of the following groups: the general population, high-risk populations (persons with valvular defects or heart problems and the immunocompromised), and pregnant women. Total medical outcomes averted for each group were calculated by using the following general equation:

Total medical cases averted = (Total adverse health outcomes caused by Q fever without PEP) – (total adverse health outcomes caused by Q fever remaining after intervention) – (cases of PEP-related adverse events). The Technical Appendix contains the equations defining each input (e.g., total adverse outcomes without PEP) of this equation.

**Figure 1:**
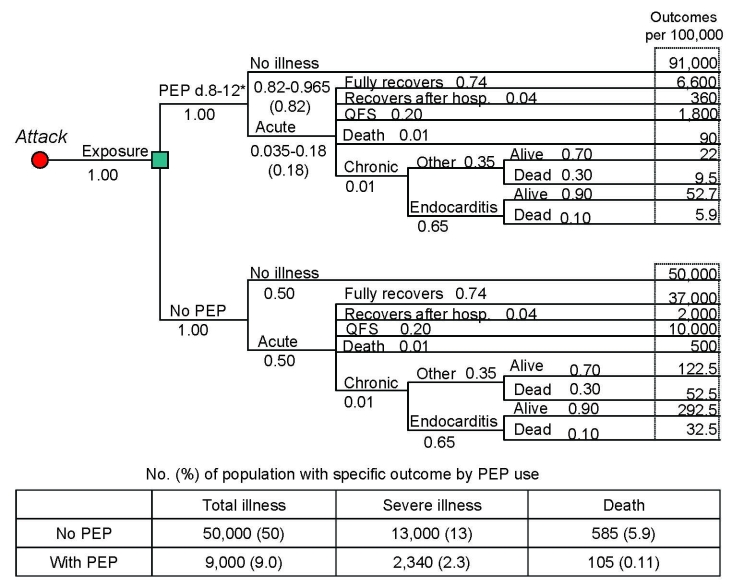
Decision tree for a general population of 100,000 based on an assumption of an aerosolized, point source, overt attack with *Coxiella burnetii* (postexposure prophylaxis [PEP] with 100 mg doxycycline 2×/d for 5 d, assuming 82% drug efficacy and 100% exposure). PEP-related adverse events are not included in this figure. The probability of each individual event occurring is provided in the decision tree under the respective event title (i.e., 1.00 for Exposure). Some events list a range of probabilities with the specific probability for this scenario in parentheses (i.e., “0.82–0.965 (0.82)” for “PEP No illness”). The number of persons with each respective outcome is listed on the right side of the tree. A summary of outcomes (total illness, severe illness and death) and the percentage of the population with such an outcome are provided in the table below the PEP and “No PEP” trees. We defined total illness as all acute illness, severe illness, and Q fever–related deaths. Severe illness was defined as hospitalization during acute infection, chronic illness, Q fever fatigue syndrome (QFS), or death. This description also applies to Figures 2 and 3.

**Figure 2:**
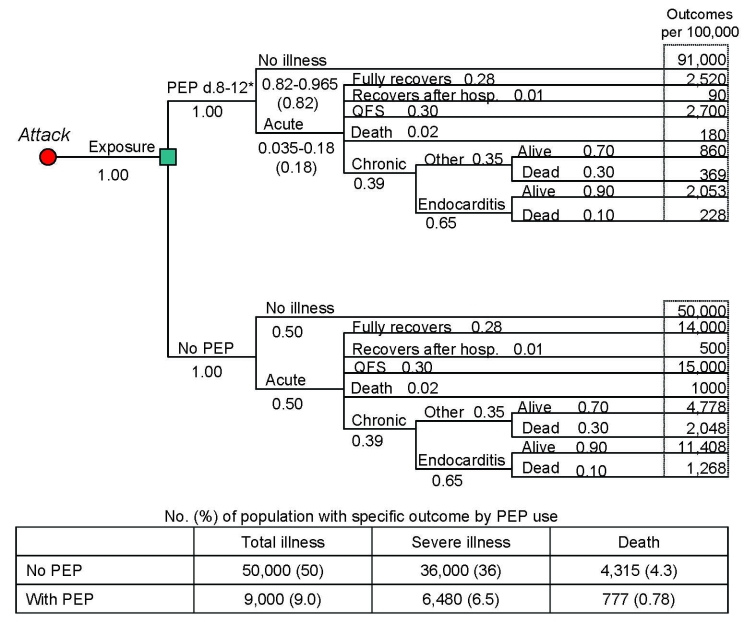
Decision tree for a high-risk population of 100,000 based on an assumption of an aerosolized, point source, overt attack with *Coxiella burnetii* (postexposure prophylaxis [PEP] with 100 mg doxycycline 2×/d for 5 d, assuming 82% drug efficacy and 100% exposure). PEP-related adverse events are not included in this figure. QFS, Q fever fatigue syndrome.

**Figure 3:**
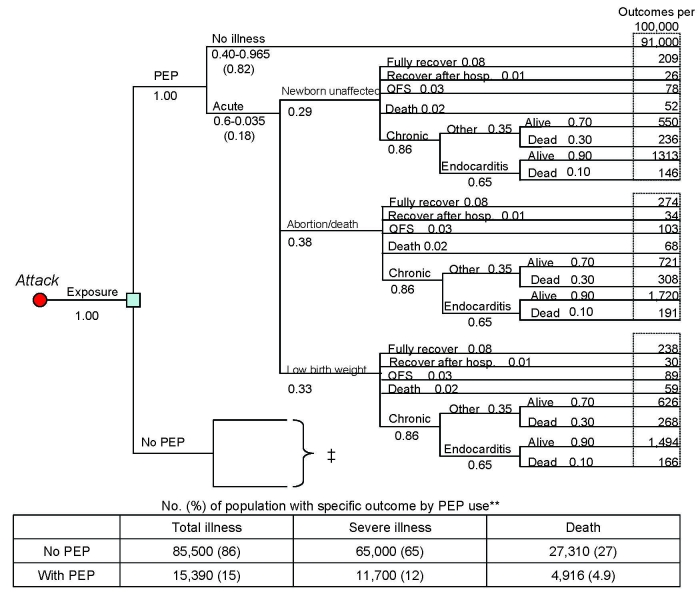
Decision tree for 100,000 pregnant women based on an assumption of an aerosolized, point source, overt attack with *Coxiella burnetii* (postexposure prophylaxis [PEP] with 160/800 mg trimethoprim-sulfamethoxazole 2×/d for duration of pregnancy, assuming 82% efficacy and 100% exposure.) PEP-related adverse events are not included in this figure. The “No PEP” segment of the tree contains the same branches and nodes as seen in the “With PEP” section, but uses different probabilities for certain variables. *The outcome of the unborn child is included in pregnant women illness estimates: low birth weight newborns were included in “Total Illness” estimates, and abortions/newborn deaths were included in all 3 outcome categories. QFS, Q fever fatigue syndrome.

To calculate adverse outcomes with and without PEP, we constructed a decision tree for each target group illustrating all possible outcomes after exposure to *C. burnetii*. The general population and high-risk populations share the same tree structure (Figures 1, 2); the tree for pregnant women incorporates the outcomes for the unborn child (Figure 3). Drug-related side effects are not included in Figures 1–3; however, the number of side effects was calculated per Equation 4 in the Technical Appendix. Total medical cases averted were calculated at 4 arbitrary levels of *C. burnetii* exposure (100%, 50%, 25%, and 10%).

### Cohort Size and Discounting

We assumed a cohort of 100,000 for each model. Also, given that each exposed patient would begin to fully experience any adverse health outcome from either Q fever or PEP within 1 year, we did not discount outcomes.

### Assumptions

Several assumptions were made in conducting this risk-benefit analysis. For simplicity’s sake, 100% compliance was assumed for persons receiving PEP. Risk-benefit analyses are based on an aerosolized, point source, overt attack, for which response can begin almost immediately. Estimates of cases and cases averted are based on the assumption that persons in whom acute or chronic illness develops receive appropriate treatment and care once a diagnosis of Q fever has been made. Those exposed received the same dose of *C. burnetii*. Although limited studies have shown an increase in dose can decrease the incubation period of the disease and/or increase the severity of illness, we were concerned with preventing illness all together (*9*,*20*).

Because Q fever has a low infectious dose (a single spore/bacterium may be enough to cause illness) (*19*), we assumed any dose would be sufficient to cause clinical infection. PEP does not affect the course or severity of illness in persons who become ill after having received prophylactic antibimicrobial drugs (persons in the PEP and no-PEP groups have the same probability of outcome events occurring once acute illness developed); persons in the No Illness group are assumed to have no latent illness.

### Interventions

Our analyses considered 2 different PEP options. For the general and high-risk populations, we assumed a PEP of 100 mg of doxycycline 2×/day for 5 days, beginning 8–12 days postexposure. As doxycycline is generally not recommended for pregnant women, we assumed a PEP of 160 mg/800 mg TMP-SMX 2×/day for the duration of the pregnancy, starting 8–12 days postexposure (*21*).

### Q Fever–related Outcomes

To provide some sense of risk-by-severity of outcome, we categorized health outcomes into 3 cumulative categories: total illness, severe illness, and death. We defined total illness as all acute illness, severe illness, and Q fever–related deaths. Severe illness includes hospitalization during acute infection, chronic illness, Q fever fatigue syndrome (QFS), and death. For pregnant women, the outcome of the unborn child is included in illness estimates: low-birthweight newborns were included in the total illness estimates, and abortions/newborn deaths were included in all 3 outcome categories.

We provide, in Table 1, the values used in the analyses based on information we obtained from an extensive literature review. The probabilities associated with each possible event were multiplied and applied to a population of 100,000 to estimate the number of people who would experience a given outcome with and without PEP (Figures 1–3). Cases averted because of PEP use were calculated (Equation 4 in the Technical Appendix).

**Table 1:** Input values used in the primary and secondary analyses of PEP efficacy*

Variable	Primary analysis (sensitivity analysis)	Sensitivity analyses		References
		Less virulent	More virulent	
Exposure	(0.10, 0.25, 0.50, 1.00)	NA	NA	NA
Efficacy of doxycycline PEP (8–12 d postexposure)	0.82 (0.82–0.965)	0.965	0.40	(*22*,*23*)
Efficacy of trimethoprim-sulfamethoxazole PEP (8–12 d postexposure)	0.82 (0.40–0.965)	0.965	0.40	(*21*,*24*,*25*)
Asymptomatic infection w/o PEP (all groups)	0.50	0.65	0.40	(*1*,*3*,*5*,*7*,*8*,*26*)
Full recovery after acute (gp)	0.74	Residual (0.934)	Residual (0.576)	(*7**–**9*)
Full recovery after acute illness (hr)	0.28	Residual (0.739)	Residual (0.076)	(*7**–**9*)
Full recovery after acute illness (pw)	0.08	Residual (0.57)	Residual (0.02)	(*7**–**9*)
Probability of hospitalization and recovery given acute illness (gp)	0.04	0.01	0.05	(*5**,**7**,**27*)
Probability of hospitalization and recovery given acute illness (hr)	0.01	0.01	0.05	(*5**,**7*)
Probability of hospitalization and recovery given acute illness (pw)	0.01	0.01	0.02	(*5**,**7*)
Q fever fatigue syndrome (gp)	0.20	0.05	0.30	(*1**,**10**,**16**,**28*–*30*)
Q fever fatigue syndrome (hr)	0.30	0.05	0.20	(*1**,**10**,**16**,**28*–*30*)
Q fever fatigue syndrome (pw)	0.03	Residual (0.03)	Residual (0.04)	(*1*,*10*,*16*,*17*,*28*–*30*)
Death from acute illness (gp)	0.01	0.001	0.024	(*7*,*9*–*11*)
Death from acute illness (hr and pw)	0.02	0.001	0.024	(*7*,*9*–*11*)
Chronic disease (gp)	0.01	0.005	0.05	(*8*,*11*,*12*,*26*,*31*)
Chronic disease (hr)	0.39	0.20	0.65	(*13*,*14*,*31*)
Chronic disease (pw)	0.86	0.39	0.90	(*17*)
Endocarditis (all groups)	0.65	0.60	0.90	(*1*,*7*,*8*)
Death from endocarditis (all groups)	0.10	0.05	0.60	(*1*,*3*,*7*,*8*,*11*,*15*,*32*)
Death from other chronic diseases (all groups)	0.30	0.05	0.60	(*9*)
Abortion or neonatal death	0.38	0.25	0.56	(*8*,*14* ,*17*,*21*)
Premature birth/low birth weight baby	0.33	0.25	0.28	(*8*,*14*,*17*,*21*)
Healthy, unaffected baby	0.29	0.50	0.16	(*8*,*14*,*17*,*21*)

*PEP, postexposure prophylaxis; NA, not applicable; gp, general population; hr, high-risk; pw, pregnant women. See online Appendix Table 1 for a detailed explanation of how the primary input values were selected.

### PEP-related Adverse Events

The medical literature was reviewed to determine the probabilities of adverse events associated with doxycycline and trimethoprim-sulfamethoxazole. Adverse events were categorized as moderate, severe, or death resulting from prophylactic antibimicrobial drug use. We assumed that a moderate PEP-related adverse event is equivalent to an acute case of Q fever, a severe PEP-related adverse event is equivalent to a severe acute case or chronic case of Q fever, and a death from PEP use is equal to a death from Q fever.

A best estimate and an upper bound estimate for the probabilities of adverse events were selected (Table 2). The best estimates for rates of AEs from doxycycline are based on estimates cited in a study of anthrax prophylaxis–related adverse events (for both doxycycline and ciprofloxacin) (*33*). The upper bound estimates for doxycycline are arbitrarily defined as 3× the best estimate probabilities. In the case of the upper bound probability of death for doxycycline, because a death rate of 0.0% was stated in the literature (*30*,*33*), 0.01% was arbitrarily assigned on the basis of the best estimate for severe adverse events (0.01%).

**Table 2:** Probability of adverse events associated with postexposure prophylactic antimicrobial agents

Level of adverse event	Doxycycline*			Trimethoprim-sulfamethoxazole†	
	Best estimate	Upper bound†		Best estimate	Upper bound
Moderate	1.01%	3.03%		3.90%	11%
Severe	0.01%	0.03%		0.00123%	0.00370%‡
Death	0.00%	0.01%		0.00037%	0.00111%

*Recommended for the general population and high-risk populations based on estimated use of 100 mg orally 2×/d for 5 d. †Recommended for pregnant women based on 160 mg/800 mg orally 2×/d for the duration of the pregnancy. ‡Arbitrary upper-bound, 3× best estimate (see text for further details).

The best estimate for moderate adverse events from TMP-SMX is based on a study that cited 3.9% (7/180) of patients discontinued antimicrobial drug treatment based on AEs (*34*). Two other studies reported that 11% of patients prematurely discontinued TMP-SMX use based on adverse events (*24*,*35*). However, these studies likely overestimate the probability of moderate AEs as some patients may discontinue use after experiencing only mild adverse events. Therefore, the lowest percent cited in the literature (3.9%) was used as the best estimate, and 11% was set as the upper bound estimate. We note that this best estimate may still be an overestimate.

A probability of 0.00037% was selected for the adverse deaths from TMP-SMX use, which is derived from a study that estimated 3.7 deaths/million treatments (*36*). We assumed that most deaths from TMP-SMX treatment are a result of toxic epidermal necrolysis (TEN) (*37*). A severe adverse event probability of 0.00123% is based on the estimate that 30% of TEN cases result in death (*37*).

As stated above, the TMP-SMX upper bound estimate for moderate adverse events was set at 11% (*24*,*35*). The TMP-SMX upper bound severe AE estimate, 0.0037%, was obtained by multiplying the TMP-SMX best estimate for severe AEs by 3. This was done to remain consistent with the arbitrary selection of an upper bound severe adverse event estimate for doxycycline, which used the same selection technique. Lastly, 0.0011% was used as the upper bound estimate for TMP-SMX-related deaths on the basis of the aforementioned assumption that 30% of severe adverse events (i.e., TEN) result in death (*37*). Because of lack of relevant data, and to avoid underestimating drug-related side effects, we assumed the upper bound estimate of doxycycline-related deaths to be ≈10× greater than that of TMP-SMX (Table 2).

### Threshold Point

The threshold point is defined as the probability of exposure to *C. burnetii* where the number of PEP-related adverse events equals the cases averted because of PEP use. The risk for adverse events equals the benefit of PEP use.

### Sensitivity Analyses

We conducted initial sensitivity analyses on the efficacy of doxycycline (96.5% and 82%) and TMP-SMX (96.5%, 82%, and 40%). These drug efficacies were chosen on the basis of a review of the literature (Table 1; Appendix Table 1, but because of lack of evidence of TMP-SMX’s efficacy as a prophylaxis for Q fever, we arbitrarily chose a low-range efficacy value (40%). Because of uncertainty in many of the input values for the primary analyses (Table 1), we conducted 2 additional scenarios labeled less virulent and more virulent. “Less virulent” and “more virulent” are the terms used to describe the lower and upper bound of the sensitivity analyses. The less virulent values are those that create a best-case scenario for health outcomes, while the more virulent analysis uses the worst-case scenario values.

As appropriate, we reduced (for less virulent) or increased (for more virulent) the input values used in the primary scenario (Table 1; Appendix Table 1). In many instances, we did not have reliable measures to define less or more virulence and values were assumed as needed. As before, we ran each of the altered virulence scenarios assuming different levels of drug efficacy (doxycycline, 82% and 96.5%; TMP-SMX, 40%, 82%, and 96.5%).

## Results

We estimate that without the use of postexposure prophylaxis, exposing a general population of 100,000 to *C. burnetii* would result in 50,000 cases of illness, 13,000 severe cases, and 585 deaths (Figure 1). Figures 2 and 3 provide results for the high-risk population and pregnant women, respectively. If we assume 82% drug efficacy for doxycycline, 9,000 cases of illness, 2,340 severe cases, and 105 deaths would occur within an exposed general population that took PEP. This translates to 41,000 cases of illness, 10,660 severe cases, and 480 deaths averted because of PEP use (82% reduction of cases). In addition, using doxycycline as PEP in a population of 100,000 (using the adverse event best estimates found in Table 2) would result in ≈1,010 moderate adverse events, 10 severe adverse events, and 0 deaths. Therefore, subtracting these adverse events from the total PEP-averted cases show that PEP use in this population would prevent 39,990 cases of total illness, 10,650 severe cases, and 480 deaths. Table 3 displays the total medical cases averted (accounting for PEP-related adverse events) for each group.

**Table 3:** Total medical cases averted because of postexposure prophylaxis*

Population	No. cases averted
General population	
All cases of illness	39,990
Severe illness	10,650
Deaths	480
High-risk population	
All cases of illness	39,990
Severe illness	29,510
Deaths	3,538
Pregnant women†	
All cases of illness	66,210
Severe illness	53,300
Deaths	22,394

*Accounting for best estimate drug-related adverse events, 82% drug efficacy, and 100% exposure. †Includes the outcome of the unborn child.

Figures 1–3 also include the percentage of each population that would develop illness/death with and without the use of PEP. Of particular importance is the probability of severe cases of illness without PEP use; 13% of the general population, 36% of the high-risk population, and 46% of pregnant women would experience severe illness. Of all cases of illness among the general population, the high-risk population and pregnant women, 26%, 72%, and 92%, respectively, would be severe. Moreover, abortion or newborn death would occur in 19% of exposed pregnant women; 16.5% would give birth to a low-birthweight baby.

**Figure 4:**
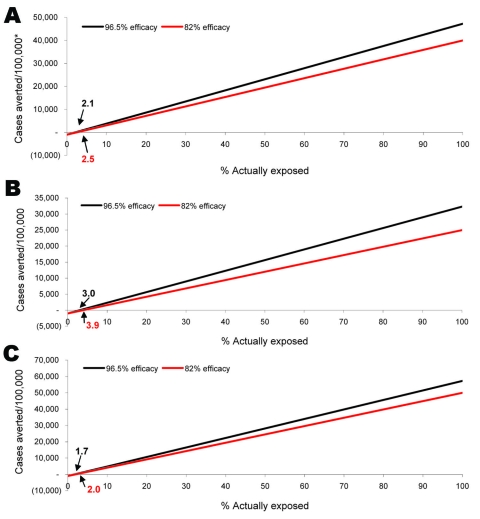
Cases of illness averted in the general population after *Coxiella burnetii* exposure with the use of postexposure prophylaxis while accounting for a 1.0% probability of adverse events, broken down by virulence scenario and drug efficacy. The “best estimate” scenario (primary analysis, A) uses best estimate input values, the “less virulent” scenario (B) uses input values that result in the least harmful outcome, the “more virulent” scenario (C) applies input values that result in the most harmful outcome or worst-case scenario. Drug efficacy refers to the efficacy of doxycycline as a post-exposure prophylaxis against *C. burnetii* infection. Analyses for doxycycline (used by the general and high-risk populations) were run at 2 potential drug efficacies: 96.5% and 82%. The threshold points, the probability of exposure where the risk of adverse events equals the risk of illness, are noted on the graphs.

The threshold point is defined as the probability of exposure to *C. burnetii* where the risk for adverse events equals the benefit of PEP use. Figure 4 illustrates the general population threshold points (run at 2 different drug efficacy values) for total cases of illness averted for the primary, less, and more virulent scenarios. The x-intercept on these graphs is the probability of exposure to *C. burnetii* at which the total number of cases of illness averted because of PEP use is equal to the number of moderate PEP-related adverse events. Therefore, for any probability of exposure greater than the stated threshold value, PEP would prevent more cases of illness than the number of adverse events PEP would cause. As Figure 4 illustrates, the less virulent or more virulent scenarios affect the estimated number of cases but do not greatly affect the threshold probabilities of exposure. For further analyses, refer to Appendix Tables 2, 3, and 4 to review univariate sensitivity analyses on various variables used in the risk/benefit scenarios. These tables show which variables have the greatest independent influence on the respective outcomes and how modifications to the input values impacts the estimated number of cases averted.

Table 4 displays all threshold points by group, outcome, drug efficacy, and probability of an adverse event (best or upper bound estimate). Threshold points (when using primary analysis input values) range from 0.0% to 7.4% for the general population and high-risk groups; the threshold points for pregnant women range from 0.001% to 32.2%.

**Table 4:** Summary of threshold points by group, drug efficacy, and probability of an adverse event (best estimate/upper bound estimate)

Population	Drug efficacy				
	96.50%	40%*	82%		
			Primary analysis	Less virulent	More virulent
General population					
All cases of illness	2.1/6.3	NA	2.5/7.4	3.88/11.65	1.98/5.94
Severe illness	0.08/0.24	NA	0.09/0.28	0.58/1.75	0.05/0.14
Death	0.00/1.8	NA	0.00/2.1	0.00/28.49	0.00/0.36
High-risk population					
All cases of illness	2.1/6.3	NA	2.5/7.4	3.88/11.65	1.98/5.94
Severe illness	0.03/0.09	NA	0.03/0.10	0.147/0.44	0.021/0.06
Death	0.00/0.24	NA	0.00/0.28	0.00/3.5	0.00/0.05
Pregnant women					
All cases of illness	4.7/13.3	11.4/32.2	5.6/15.7	9.99/28.2	4.16/11.7
Severe illness	0.002/0.006	0.005/0.014	0.002/0.007	0.0069/0.021	0.0016/0.0047
Death	0.001/0.004	0.003/0.010	0.002/0.005	0.0053/0.0156	0.0006/0.0019

*Doxycycline was not evaluated at 40% drug efficacy. NA, not available.

## Discussion

Based on this study, we believe many cases of illness and deaths could be prevented with the use of PEP after a deliberate, overt release of *C. burnetii*. Without taking social or political concerns into account, a threshold point can be interpreted as the decision point for PEP use. Any value above the threshold point indicates that the benefits of PEP use outweigh the risks for adverse events, therefore implying PEP should be recommended for any probability of exposure to *C. burnetii* above the stated threshold point. For the general and high-risk population, when doxycycline is used as a postexposure prophylactic antimicrobial drug, due to low rates of AE, the argument to administer PEP in most cases of potential exposure is strong. Even in the worst case scenario (upper bound adverse event estimate), the threshold point for total illness is relatively low at 7.4%.

Use of TMP-SMX for pregnant women also favors PEP use in most scenarios. Due to higher rates of moderate adverse events among TMP-SMX users, the threshold point for total illness is not as low as seen for doxycycline users (the general population and high-risk populations), but TMP-SMX threshold points still exhibit the importance of providing prophylaxis. Although the upper bound AE estimate in conjunction with the lower bound estimate of drug efficacy (40%) indicates 32.2% is the threshold point for total illness, this is the worst case scenario. Both the upper bound estimate for adverse events and the upper bound drug efficacy are considered to be overestimations to the preferred best estimate. The efficacy of TMP-SMX as a form of PEP is likely closer to 82% on the basis of its efficacy as a prophylaxis for several infections such as toxoplasmosis and *Pneumocystis carinii* pneumonia (*25*). Moreover, because Q fever is primarily an incapacitating agent, severe illness is likely a good proxy for the disease’s effects on a population. Therefore, the severe case threshold point (assuming 82% drug efficacy) is low, lying between 0.002% and 0.007% for pregnant women, which provides strong support for PEP use in most cases where exposure is suspected.

On the basis of these analyses, we determined there are 2 variables that most strongly influence the model. First, the efficacy of the drug as prophylaxis for Q fever is 1 of the most important variables in this model. Understandably, if the antimicrobial agent is effective, considerable illness and death will be prevented. Unfortunately, there are limited data on the efficacy of these drugs at preventing Q fever illness. Sensitivity analysis was conducted on this variable to account for this uncertainty; however, based on treatment experiences with these drugs (doxycycline’s efficacy ranges from 82% to 99% for *Chlamydia trachomatis* cervicitis) (*22*,*23*), we think the best estimates used in this study (82%) are conservatively close to the actual drug efficacies. A second important variable in the model is the probability of PEP-related adverse events. Once again, attempts were made to account for limited data by providing best and upper bound estimates for adverse events.

Although this risk-benefit analysis may be very useful when developing policy and official PEP recommendations, there are limitations to this design, such as some of the data on which this analysis is based. Particularly, the recommendation that PEP should be administered 8–12 days postexposure is based on a single study conducted on only 5 persons and 1 type of antimicrobial drug (oxytetracycline). We acknowledge that these are limited data, but administration 8–12 days postexposure still remains the official recommendation of the US Army Medical Research Institute of Infectious Diseases. As a result, we remain consistent with current recommendations, but we are open to alternative options if more evidence becomes available. Conducting further animal studies would help to clarify the optimal time and duration of drug administration and the ideal antimicrobial drug. Newer drugs are now available and these may be more effective at preventing illness.

Another limiting variable was the death rate from acute Q fever infection among non-PEP users. Our study assumed that all persons in whom acute or chronic illness develops are assumed to receive appropriate treatment and care for the duration of illness once a diagnosis of Q fever has been made. Although no estimates are available in the literature for the death rate among treated persons (only untreated), we chose to use the death rate for untreated persons (1%). However, because of the uncertainty of this value, sensitivity analyses were conducted to assess variable effect on the number of severe cases of illness averted. Appendix Table 4 shows how the change in input values for both the PEP and no PEP groups does not greatly impact the total number of severe cases of illness averted within the general population.

Also, as mentioned before, this risk-benefit analysis is based on several assumptions, such as an overt attack, 100% exposure, and 100% compliance of the entire study population. These assumptions simplify the situation and create a more quantifiable, but more unnatural, scenario. Although an overt attack is less likely to occur, future models can adjust this assumption to account for a delay in diagnosis or outbreak detection. Prophylactic efficacy and ultimately the number of illnesses/deaths could vary depending on such factors as compliance and the number of organisms to which the person was exposed. Another limitation to acknowledge is that *C. burnetii* is very resilient in the environment and exposure a long time after the initial dispersal could be an issue. Our model has only accounted for a single-exposure event, but future models should address this point.

Several issues will also be important when considering PEP recommendations. First, when selecting a representative threshold value for each risk group, social and political concerns must be acknowledged and considered. If a threshold value is 2.5%, it may be more realistic for decision makers to instruct all persons with any probability of exposure to take PEP rather than use valuable time and resources to determine a person’s numerical probability of exposure. In addition, before providing a specific numeric threshold value in the guidelines, knowing how to measure that probability of exposure is important. For example, if 7% is provided as a threshold, there must be a mechanism for differentiating between 6% and 8% or 5% and 15%. These threshold points and PEP recommendations must be useful and realistic. Some research has been conducted to evaluate how to determine likely concentrations of a bioterrorism agent and a person’s level of exposure by using computer modeling and simulation (*38*). Further studies on the assessment of exposure would be beneficial; modeling Q fever exposure would be especially critical given *C. burnetii*’s low infectious dose and high environmental stability (*9*).

Also, this study does not directly address children <8 years of age. In general, this population is not at higher risk for illness/death from Q fever infection than the general population. However, risk-benefit analyses for children should be conducted to provide guidance on PEP recommendations for this age group. Lastly, this analysis was conducted on the basis of the most frequently suggested prophylaxis regimens. However, other antimicrobial drugs should be evaluated with risk-benefit analytic methods.

Cost was not considered in this risk-benefit analysis. Further studies are warranted to expand and support various aspects of this analysis, including estimating the cost associated with the use of PEP after a deliberate release of *C. burnetii*.

This study illustrates the importance and benefit of postexposure prophylaxis in a mass-exposure scenario and also weighs the risk for prophylaxis-related adverse events. Early identification of persons at increased risk for Q fever illness (pregnant women and high-risk populations) would be crucial in providing proper PEP and, in turn, preventing illness/death in these groups. Based on the study assumptions of exposure and compliance, PEP may be warranted and is likely to be effective at averting cases of illness and deaths in all 3 population groups when the probability of exposure to *C. burnetii* is above the population-specific threshold point.

## Supplementary Material

Appendix Table 1 Explanation, rationale, and comments on the primary analysis input values used in the study*

Appendix Table 2 Univariate sensitivity analyses of the input values for the general population variables on severe cases of illness averted due to PEP, accounting for best estimate drug-related adverse events and 100% exposure*

Appendix Table 3 Univariate sensitivity analysis of the chronic disease variable for pregnant women assessing the impact of this variable on the cases of severe illness averted due to postexposure prophylaxis

Appendix Table 4 Univariate sensitivity analyses of the death from acute illness variable for the general population assessing the impact of this variable on the cases of severe illness averted by use of PEP*

Technical Appendix Prophylaxis after Exposure to Coxiella burnetii
