# Supplementary material for: Prophylaxis after Exposure to Coxiella burnetii
Source: Emerg Infect Dis. 2008 Oct;14(10):1558–66. doi: 10.3201/eid1410.080576 (PMC2609859; doi:10.3201/eid1410.080576)
Supplement: Appendix Table 1 — Explanation, rationale, and comments on the primary analysis input values used in the study* [file 08-0576_appT1-s1.pdf]

Appendix Table 1. Explanation, rationale, and comments on the primary analysis input values used in the study*

Variable	Primary analysis (Sensitivity analysis)	Explanation, rationale and comments
Exposure	(0.10, 0.25, 0.50, 1.00)	A range representing the percent of people actually exposed to *Coxiella burnetii*
Efficacy of doxycycline as PEP (8–12 d postexposure)	0.82 (0.82–0.965)	No specific estimates are available for doxycycline's efficacy as PEP for Q fever. These numbers are based on studies of doxycycline as a treatment for *Chlamydia trachomatis* infections, which resemble *C. burnetii* in several ways (*22,23*)
Efficacy of trimethoprim-sulfamethoxazole as PEP (8–12 d postexposure)	0.82 (0.40–0.965)	No specific estimates are available for TMP-SMX efficacy as PEP for Q fever. For comparison's sake, we chose similar estimates to doxycycline, but added a lower-bound estimate of 40%. However, the efficacy is likely higher than that, based on TMP-SMX's efficacy with other bacterial infections. Among HIV-positive patients, the infection rate per 100 patient-years of follow-up was 31 for any bacterial infection (*25*). For an intent-to-treat population, TMP-SMX had an 87% efficacy. (*24*)
Asymptomatic infection w/o PEP (all groups)	0.50	Numerous sources state ≈50% (>50, <60%) of Q fever infections remain asymptomatic (*1,3,5,7,8,25*)
Full recovery after acute (gp)	0.74	Most acute cases result in a full recovery (*7–9*). Based on the probabilities of the other possible acute outcomes, 74% of the total acute cases result in full recovery of the patient (residual).
Full recovery after acute illness (hr)	0.28	Most acute cases result in a full recovery (*7–9*). Based on the probabilities of the other possible acute outcomes for high-risk populations, 28% of the total acute cases result in full recovery of the patient (residual).
Full recovery after acute illness (pw)	0.08	Based on the probabilities of the other possible acute outcomes for pregnant women, 8% of the total acute cases result in full recovery of the patient (residual).
Recovery from acute illness after hospitalization (gp)	0.04	5% of all acute, symptomatic Q fever cases require hospitalization (*5,7*). 2% of Q fever pneumonia cases require admission to the intensive care unit (*5,7,27*). As some of these hospitalizations would occur among chronic disease cases and patients who die, this 5% is applied to the population acute case-patients (78%) who will eventually fully recover from acute illness. Therefore, 4% of acute case-patients will be hospitalized at some point during their illness but will still have a full recovery.
Recovery from acute illness after hospitalization (hr)	0.01	5% of all acute, symptomatic Q fever cases require hospitalization (*5,7*). Therefore, when 5% is applied to the population of acute cases that eventually recovers (29%), it is estimated that 1% of all acute case-patients will fully recover after being hospitalized.
Recovery from acute illness after hospitalization (pw)	0.01	Because of the vulnerability of pregnant women to Q fever and the likelihood that these women would be closely observed if acutely ill, the percentage of recovering, acute case-patients requiring hospitalization is based on the high-risk population's percentage (1%).
Q fever fatigue syndrome (gp)	0.20	Studies cite 10%–30% of acute cases develop QFS, with the largest studies citing between 20%–30% (*1,10,16,30,28,29*) A low/mid-range value was used for the general population as a conservative estimate.
Q fever fatigue syndrome (hr)	0.30	QFS develops in 10%–30% of acute cases; 30% was selected as the value for high-risk populations because they would likely be more susceptible to QFS and other chronic conditions because of their immunocompromised state and/or the presence of a heart defect.
Q fever fatigue syndrome (pw)	0.03	No study cites the proportion of pregnant women in whom QFS develops. However, based on 86% of acute case-patients developing chronic illness (*17*); 12% will not advance to chronic illness. Given that QFS would develop in 20% of this population (see QFS above), QFS will develop in 3% of all acutely ill pregnant case-patients
Death from acute illness (gp)	0.01	Most studies cite a mortality rate of ≈1% from acute Q fever when left untreated (range 0.5%–2.4%) (*9–11*). A mortality rate of 1% was used in this analysis for the general population as this is the most consistently cited value and on the lower bound of the estimates. No studies specifically state the mortality rate when treatment is given; however, for treatment to be effective, it must be administered within 3 d of illness (7). Therefore, the mortality rate may not be extremely different between treated and untreated unless the antimicrobial drugs are given in the early stages of illness.
Death from acute illness (hr)	0.02	The upper bound of mortality estimates (see above) was used because this population is more vulnerable to severe disease and death.
Death from acute illness (pw)	0.02	The upper bound of mortality estimates (see above) was used because this population is more vulnerable to severe disease and therefore death.
Chronic disease (gp)	0.01	Sources indicated that chronic illness develops in <1%–5% of all patients with acute cases (*8,11,12,26,31*). A conservative estimate was used here.
Chronic disease (hr)	0.39	Even with treatment, chronic disease develops in 39% of persons with valvular defects and acute Q fever (if Q fever is untreated, chronic disease

		develops in 75%) (*13,14*). HIV-positive persons were 13× more likely than HIV-negative individuals to develop chronic illness (*31*).
Chronic disease (pw)	0.86	Raoult et al. report that 86% (12/14 cases) of pregnant women who were diagnosed with acute Q fever went on to develop chronic illness (*17*). This may be an overestimate, due to the small sample size and identification of the more severe acute cases of Q fever. However, it is the best available estimate.
Endocarditis (all groups)	0.65	60%–73% of all chronic Q fever infections are endocarditis (mode: 65%) (*1,7,8*).
Death from endocarditis (all groups)	0.10	Several sources agree that the death rate among treated Q fever endocarditis patients is ≈10% (*1,3,7,8*). This rate increases to 30%–60% if endocarditis is left untreated (*11,15,32*).
Death from other chronic diseases (all groups)	0.30	Bossi et al. state that the death rate for all chronic infections is between 30% and 60% (*9*) Although not stated directly in the article, it is assumed that this range is dependent on the type of chronic illness and whether treatment was administered appropriately. The conservative estimate (30%) was used in this analysis based on the assumption that chronic cases would be identified and treated properly.
Abortion or neonatal death	0.38	Little data is available on pregnant women, but Raoult describes 24 cases of women who contract Q fever during pregnancy and were identified during the acute stage of illness (resulting in 38% abortions, 33% premature births, 29% w/o abnormalities) (*17*). A previous Raoult study of 32 acute cases among pregnant women showed the following breakdown: 56% abortions/neonatal deaths, 28% premature births, 16% normal births (*8*). A Maltezou study states that 86% of pregnancies are complicated (*14*). Although these percentages are likely overestimations of negative, fetal outcomes (the most serious cases having been identified), they are the best estimates available at this time.
Premature birth/low birth weight baby	0.33	
Healthy, unaffected baby	0.29	

*PEP, postexposure prophylaxis; gp, general population; hr, high-risk; pw, pregnant women; TMP-SMX, trimethoprim-sulfamethoxazole; QFS, Q fever chronic fatigue syndrome.
