# Supplementary material for: Prophylaxis after Exposure to Coxiella burnetii
Source: Emerg Infect Dis. 2008 Oct;14(10):1558–66. doi: 10.3201/eid1410.080576 (PMC2609859; doi:10.3201/eid1410.080576)
Supplement: Appendix Table 2 — Univariate sensitivity analyses of the input values for the general population variables on severe cases of illness averted due to PEP, accounting for best estimate drug-related adverse events and 100% exposure* [file 08-0576_appT2-s2.pdf]

Appendix Table 2. Univariate sensitivity analyses of the input values for the general population variables on severe cases of illness averted due to PEP, accounting for best estimate drug-related adverse events and 100% exposure*

Variable (only for general population)	(Primary value)	Severe cases of illness averted (lower bound analysis)	Severe cases of illness averted (upper bound analysis)
Efficacy of doxycycline PEP, 8–12 days postexposure	(0.82)	5,200 (0.40)	12,545 (0.965)
Asymptomatic infection w/o PEP	(0.50)	13,260 (0.40)	6,760 (0.65)
Probability of hospitalization and recovery given acute illness	(0.04)	9,430 (0.01)	11,070 (0.05)
Q fever fatigue syndrome	(0.20)	4,510 (0.05)	14,760 (0.30)
Death from acute illness	(0.01)	10,291 (0.001)	11,234 (0.024)
Chronic disease	(0.01)	10,455 (0.005)	12,300 (0.05)
Endocarditis	(0.65)	10,660 (0.60)	10,660 (0.90)
Severe cases of illness averted (primary analysis)	10,660		

*PEP, postexposure prophylaxis.
