# Supplementary material for: Prophylaxis after Exposure to Coxiella burnetii
Source: Emerg Infect Dis. 2008 Oct;14(10):1558–66. doi: 10.3201/eid1410.080576 (PMC2609859; doi:10.3201/eid1410.080576)
Supplement: Appendix Table 3 — Univariate sensitivity analysis of the chronic disease variable for pregnant women assessing the impact of this variable on the cases of severe illness averted due to postexposure prophylaxis [file 08-0576_appT3-s3.pdf]

Appendix Table 3. Univariate sensitivity analysis of the chronic disease variable for pregnant women assessing the impact of this variable on the cases of severe illness averted due to postexposure prophylaxis

Variable (only for pregnant women)	(Primary value)	Severe cases of illness averted (lower bound analysis)	Severe cases of illness averted (upper bound analysis)
Chronic disease	53,300 (0.86)	34,030 (0.39)	54,940 (0.90)
