# Supplementary material for: Prophylaxis after Exposure to Coxiella burnetii
Source: Emerg Infect Dis. 2008 Oct;14(10):1558–66. doi: 10.3201/eid1410.080576 (PMC2609859; doi:10.3201/eid1410.080576)
Supplement: Appendix Table 4 — Univariate sensitivity analyses of the death from acute illness variable for the general population assessing the impact of this variable on the cases of severe illness averted by use of PEP* [file 08-0576_appT4-s4.pdf]

Appendix Table 4. Univariate sensitivity analyses of the death from acute illness variable for the general population assessing the impact of this variable on the cases of severe illness averted by use of PEP*

Data	Analysis 1	Analysis 2	Analysis 3	Analysis 4	Analysis 5	Analysis 6	Analysis 7
Value of death from acute Illness for PEP group	0.01	0.001	0.024	0.005	0.002	0.0005	0.01
Value of death from acute illness for no PEP group	0.01	0.001	0.024	0.01	0.01	0.001	0.005
Cases of severe illness averted w/PEP use	10,660	10,291	11,234	10,705	10,732	10,296	10,410

*PEP, postexposure prophylaxis.
