# Supplementary material for: Prophylaxis after Exposure to Coxiella burnetii
Source: Emerg Infect Dis. 2008 Oct;14(10):1558–66. doi: 10.3201/eid1410.080576 (PMC2609859; doi:10.3201/eid1410.080576)
Supplement: Technical Appendix — Prophylaxis after Exposure to Coxiella burnetii [file 08-0576_Techapp-s5.pdf]

Prophylaxis after Exposure to *Coxiella burnetii*

Technical Appendix

Equation 1: (in the main text), the inputs (on the right side of equation) are calculated as follows.

For each outcome (i.e., branch of tree–see Figures 1–3 in text):

Equation 2: Number of a given adverse health outcome without PEP = Number persons exposed × probability of given outcome

Equation 3: Number of a given adverse health outcome after PEP = Number persons exposed × probability of outcome × effectiveness of PEP against given outcome

For each type of PEP-related adverse health outcome (see Table 2 in text).

Equation 4: Number of given PEP-related adverse events = number administered PEP × probability of given adverse event
